# Correlates between spirituality, self-regulation and mental health in youths from the community: A micro-longitudinal study protocol

**DOI:** 10.1136/bmjopen-2026-116754

**Published:** 2026-11-11

**Authors:** Sebastien Urben, Chloé von Niederhäusern, Setareh Ranjbar, Kerstin Jessica Plessen, Jennifer Glaus

**Affiliations:** 1 Division of Child and Adolescent Psychiatry, Lausanne University Hospital, Lausanne, Switzerland; 2 Center of Psychiatric Epidemiology and Psychopathology, Lausanne University Hospital, Lausanne, Switzerland

**Keywords:** MENTAL HEALTH, Quality of Life, Child & adolescent psychiatry

## Abstract

**Abstract:**

**Background and objective:**

Adolescence and young adulthood represent critical developmental stages during which mental disorders often emerge, with the potential to impede perceived quality of life. Spirituality (i.e., the search for the sacred) and self-regulation (i.e., intrinsic processes regulating emotions, thoughts and behaviours) are recognised as protective factors for mental health. However, their dynamic interplay remains largely unexplored, particularly in real life and in real time among youths. This study, developed with the help of young partners, addresses this gap by investigating the longitudinal associations between spirituality, self-regulation and mental health using an ecological momentary assessment (EMA) approach.

**Methods and analysis:**

We plan to recruit 120 adolescents and young adults (aged 16–20, expected attrition rate of 20%) from the community to complete a qualitative semi-structured interview assessing their beliefs, spiritual or religious activities, role models and ‘meaning in life’. In addition, participants will take part in a multi-wave intensive longitudinal study. Trait-level assessments will be conducted at two time points, 3 months apart, to capture between-person differences. Additionally, to assess within-person dynamics, participants will complete EMA surveys four times daily over 10 consecutive days in two waves, also 3 months apart. Measures will include facets of spirituality (e.g., beliefs, meaning, collective consciousness), self-regulation (e.g., self-control, emotional regulation, impulsivity), as well as mental health indicators (emotional and behavioural symptoms) and quality of life. Qualitative data will be analysed through a reflexive thematic analysis method, whereas quantitative associations will be assessed using linear mixed models and dynamic network analyses.

**Ethics and dissemination:**

We obtained approval from the Ethics Committee of the Vaud Canton, Switzerland, before data collection began in May 2025. Findings will be disseminated through open access peer-reviewed journals, conferences on adolescent mental health and shared with practitioners, educators and youth organisations. Results will also be made accessible to the general public. This study aims to inform personalised preventive and therapeutic interventions by elucidating real-time mechanisms linking spirituality, self-regulation and mental health in youths.

**STRENGTHS AND LIMITATIONS OF THIS STUDY**

- This study considers qualitative and quantitative approaches.
- We use real-time, multi-wave ecological momentary assessment to examine components of spirituality, self-regulation and mental health in youths.
- We explain both within-person and between-person variations, providing a fine-grained understanding of the temporality and dynamics of these processes.
- The age range is restricted to 16–20 years, thus, the results may not be generalisable to younger adolescents or older adults.
- Reliance solely on self-reported data may introduce bias (e.g., social desirability, subjective interpretation).

## Introduction

### Integrative approach of mental health

 Adolescence or emerging adulthood is a critical and vulnerable period when the most serious mental disorders typically emerge.^1^ This is a period of transition during which problems of mental health may arise, potentially resulting in a perceived decline in overall quality of life (QoL).^2^

In this context, an integrative and holistic approach to the mental health of youths allows to focus on care and self-care putting forward the psychological, physical and spiritual needs of the individual.^3^ From this perspective, a biopsychosocial approach enriched by the inclusion of spirituality is crucial for understanding both somatic^4^ and mental health.^5^ This is in line with a recent Swiss population-based survey,^6^ showing that 79% of the respondents would like their healthcare professional to use an integrative and holistic approach to meet their healthcare needs. Similarly, in 2019, a Swiss national survey showed that 40% of people believe in a single God and 51% believe that a higher force guides our destiny.^7^ Moreover, recent data from the Swiss Federal Statistical Office in 2024 indicate that 56.1% of respondents consider religion and spirituality important during challenging life situations, and 49.1% of individuals aged 15–24 engage in prayer practices.^8^

Mental health should not be viewed solely as the absence of psychopathology,^9^ but rather as a multi-dimensional construct encompassing both well-being and psychological difficulties. Specifically, mental health can be conceptualised as consisting of two related yet distinct dimensions: well-being, including positive mental health (PMH) or psychological QoL and psychopathology.^10 11^ Given that most studies have focused primarily on psychopathological symptoms, the present study adopts a more comprehensive approach by examining the positive dimensions of youth mental health.

### Spirituality

We defined spirituality as “the search for the sacred,”^12^ with the sacred relating to “aspects of life that are perceived as manifestations of the divine or imbued with divine-like qualities, such as transcendence, immanence, boundlessness and ultimacy.”^12^ Thus, spirituality refers to a process allowing people to discover what is of ultimate importance to them in life. It has personal, interpersonal and transpersonal dimensions consisting, mainly of four components^13^: (a) a belief in a higher power or universal intelligence; (b) an inner search for meaning and purpose (i.e, self-discovery process); (c) authentic connections to others based on deep respect and reverence for life and (d) an eco-awareness which represents a connection to nature, respect for the environment, and the belief that the Earth is sacred. Thus, spirituality is treated as a broader construct encompassing transcendence, connectedness and existential orientation, whereas meaning in life is treated as a specific psychological construct referring to the perceived presence of meaning and the search for meaning.^14–18^

Spirituality includes, but is not limited to, religion or religiosity. Indeed, religiosity refers to narrower concepts that consider practices, beliefs and symbols organised into a system of faith.^19^ It is important to note that spirituality and religiosity have different impacts on individuals. Religiosity appears to be beneficial in the adoption of better lifestyle habits, including diet, tobacco and alcohol consumption. Thus, the effects of religiosity on health may be attributed to the behavioural regulation of health habits.^20^ In contrast, spirituality has been linked to an increased sense of meaning, purpose, resilience, satisfaction, happiness and well-being,^21–23^ and provides meaning and support in times of stress.^24–26^ In particular, spirituality has been associated with biomarkers of health and somatic disease progression, such as blood pressure, cardiac reactivity and immune system markers.^20^ Therefore, systematic reviews and meta-analyses demonstrated that spirituality represents a protective factor for mental health^27–32^ especially in youths.^24 33^

Building on this multi-dimensional view of spirituality, it has been proposed^34^ that religious traditions and spirituality provide five complementary psychological resources that support coping and meaning-making: (1) emotional–affective: experiences of transcendence and emotional regulation; (2) psychosocial: social support and belonging; (3) cognitive: coherent worldviews for interpreting life events; (4) ethical: moral values and behavioural guidelines for self-regulation and (5) identity: roles and exemplars that help individuals situate themselves in the world. Together, these resources form a flexible constellation that individuals can draw on according to their needs, offering a comprehensive framework for understanding how spirituality promotes well-being.

Evidence suggests that the impact of spirituality on health is primarily mediated through physiological processes sustaining emotion regulation, or more largely self-regulation.^20^ Self-regulation is an umbrella term for intrinsic mechanisms that govern emotions, thoughts and behaviours, often aligning immediate actions with long-term goals,^35^ and it constitutes a critical protective factor for mental health. Among Indian and Emirati adolescents, self-control (a key aspect of self-regulation) has been shown to moderate the relationship between spirituality and internalised symptoms, as well as life satisfaction (or QoL).^36^

### Qualitative approach

Qualitative studies on youth spirituality have mainly evolved along two lines: clinical/health-related settings and cultural environments where religion and spirituality are particularly salient. In health-related studies, spirituality is often conceptualised as a coping resource, particularly through hope, as illustrated by qualitative work with adolescents and young adults living with cancer^37^ and by studies linking spiritual health to hope among adolescents.^38^ More exploratory studies have also examined how adolescents define spirituality and what shapes these meanings (e.g., purpose, relationships, values, everyday practices); nevertheless, all existing studies have been conducted in contexts where religion or spirituality holds a more specific culturally prominent role compared with European countries.^38–40^

In Switzerland, qualitative studies have largely focused on specific groups. For example, research with Muslim and Buddhist youths highlights religious individualisation and efforts towards social recognition and civic participation.^41^ In addition, qualitative work with Swiss secular individuals (ie, individuals without religious affiliation) further shows that spirituality can be articulated beyond religious affiliation as a multidimensional construct.^42^ However, to the best of our knowledge, the literature lacks qualitative evidence on how spirituality is experienced and interpreted in the everyday lives of Swiss adolescents from the general population.

### Ecological momentary assessment

Moreover, previous knowledge of the association between spirituality, self-regulation and mental health has been mainly developed through quantitative assessments with poor ecological validity, cross-sectional design and lack of attention to within-person variations. Ecological momentary assessment (EMA), which refers to frequent and repeated measurements in real life and in real time,^43^ enables the examination of the dynamic interrelationships between spirituality, self-regulation and mental health. In this perspective, EMA has been used to study spirituality,^44–52^ mainly among American participants and in adult community samples.^45–50 52^ For instance, the SoulPulse project^45–49^ had a major impact on the study of spirituality in real time by examining daily spiritual experiences among adults. Indeed, this project observed that daily spirituality served as a buffer against daily stressors,^47^ moderated the relationship between stressful life events and well-being,^46^ increased well-being both at the state and trait level^46^ and promoted feelings of love and care for others in stressful situations.^45^ Awareness of a higher being in everyday life was also observed^48^ to precede feelings of meaningfulness.^49^ The only non-American EMA study on spirituality was conducted in a Dutch clinical sample of adults and found that affective representations of God and spiritual experiences varied significantly within the day.^51^ Only one EMA study^44^ in the USA had sampled adolescents and found that after Ramadan, adolescents felt more connected to Allah in their daily lives and had a lasting effect toward more self-control and patience.^44^ In this line, daily diary studies involving adolescents with cystic fibrosis found that engagement in spirituality resulted in greater treatment adherence^53^ and that imbuing their own body with divine significance (i.e., body sanctification) led to better sleep hygiene and other health-protective behaviours.^54^

### Research gaps

The literature on qualitative studies of youths’ spirituality remains scarce and has predominantly focused on highly religious or clinical populations, such as adolescents with chronic illnesses^55^ or those living in strongly religious cultural contexts.^38–40^ While these studies provide important insights into how young people understand spirituality, they do not address the everyday spiritual experiences of youths from the general population in secular European settings. As a result, the field still lacks systematic qualitative accounts of how young people experience, articulate and interpret spirituality in their daily lives. This gap is especially salient in Switzerland, where a culturally diverse religious and spiritual environment may shape both the content of spirituality and its associations with well-being, calling for qualitative inquiry that is sensitive to contextual and multi-dimensional aspects of lived experience.^56 57^

In addition, the existing literature does not, to the best of our knowledge, combine such qualitative perspectives with intensive quantitative assessments; this design is therefore well suited to clarify how spirituality relates to self-regulation processes and mental health in the natural environments of Swiss youths. In this perspective, the use of multiple waves of EMA contributes to the accuracy and diversity of daily observations by capturing different and distant slices of life.^43^ However, all previous studies had a single-wave EMA approach^45–52^ except the Ramadan study, which used a three-wave EMA.^44^ Therefore, intensive longitudinal studies are needed to provide additional evidence and a greater degree of proof of spirituality as a protective factor in real life and real time among youths from the general population. It is important to note that these concepts encompass various components that have not been thoroughly explored until now. All the mentioned EMA studies show a predominant focus on adults and on the American population. Importantly, none of these studies evaluated spirituality, self-regulation and mental health together and across their different components. Therefore, the understanding of the real-time dynamic associations between the various facets of spirituality, self-regulation and mental health as well as QoL at the within-person level and in a real-life context is still largely unexplored in European adolescent and young adult populations. Furthermore, no studies exist in Switzerland, yet, despite the fact that the majority of the Swiss population^6^ would like their healthcare professionals to take this aspect into account.

### The current research project

Our overall objective is to gain a deeper understanding of how the components of spirituality and self-regulation impact adolescent and young adult mental health in real life and in real time. More specifically, we expect to gain important insights at both the between-person and within-person levels, into the specific roles played by distinct components of spirituality and self-regulation in their associations with the mental health and QoL of youths.

In particular, the aim of this study is to gain a deeper understanding of youths mental health by exploring the qualitative, as well as the quantitative, dynamic and longitudinal associations of different facets of spirituality, encompassing the emotional–affective, psychosocial, cognitive, ethical and identity-related dimensions, in conjunction with the components of self-regulation (e.g., self-control, emotional regulation, impulsivity, sense of self-efficacy), mental health (e.g., psychopathological symptoms) and QoL.

Given the limited empirical evidence on the combined role of spirituality and self-regulation in youth mental health, the present study is primarily exploratory. Nevertheless, based on the few previous research, we might broadly expect that greater self-regulatory capacities would be associated with better mental health outcomes and that higher levels of spirituality, particularly the presence of meaning in life, would be positively related to PMH and QoL. The specific contributions and the role (i.e., mediating or moderating effect) of the different dimensions of spirituality and self-regulation will be explored without a priori hypotheses.

## Methods and analysis

This study combines qualitative interviews alongside quantitative measures through a two-wave intensive-longitudinal measures. We plan to recruit adolescents and young adults in the community of the Lausanne region in the French-speaking part of Switzerland. The data collection phase finished in March 2026.

### Participants

We will recruit 120 participants aged between 16 and 20 years. The inclusion and exclusion criteria are described in table 1.

**Table 1:** Inclusion and exclusion criteria for participants

Criteria	
Inclusion	Speaking fluent French
	Having a smartphone with access to internet data and a Swiss phone number
	Being aged between 16 and 20 years
	Written informed consent
	Known significant difficulties in intellectual, conceptual, practical or social adjustment that indicate potential mild or more severe disorders of intellectual development (International Classification of Diseases ICD, 11th revision (ICD-11), 06A00)^85 86^
Exclusion	Known significant difficulties in reading that indicate a potential developmental learning disorder with impairment in reading (ICD-11, 6A03.0)
	Known major life crisis (e.g, active suicidal behaviours or thoughts, acute or florid psychosis)
	Known significant difficulties in intellectual, conceptual, practical or social adjustment that indicate a potential mild or more severe disorders of intellectual development (ICD-11, 06A00)^85 86^

Participants with current or past psychiatric disorders were eligible for inclusion. Consistent with the study’s objective of examining mental health across the continuum from well-being to psychological difficulties, no exclusion criteria related to psychiatric diagnoses were applied.

### Procedure

The study will be conducted in five main steps: (1) qualitative interview; (2) online baseline measures; (3) a 10-day period of EMA; (4) a 3-month follow-up assessment and (5) a second 10-day period of EMA.

The protocol procedure is summarised in figure 1.

**Figure 1:**
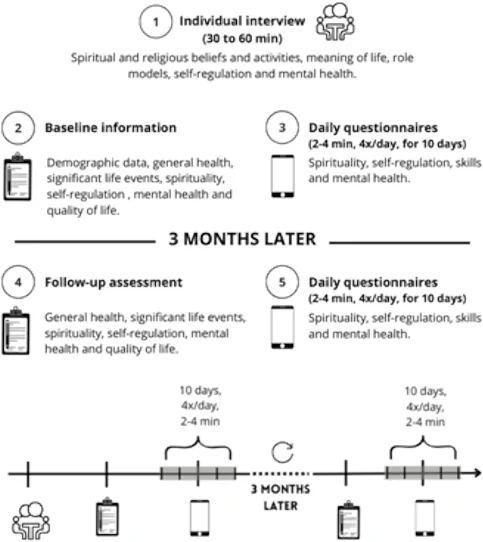
Study procedure.

### Measures

#### Qualitative assessment

We will conduct a qualitative semi-structured interview (either face-to-face or via Webex; see in online supplemental file 1). We will ask participants to talk about their beliefs, spiritual or religious activities, role models and meaning in life. This interview will last for 30 min to one hour. The interview will be recorded for analysis purposes. The verbatim will be transcribed and double-checked for quality. The interviewer will be a psychologist or a master’s student in psychology. The main themes that will be addressed during the interview are related to spirituality and resources the participants use to cope with their everyday life and to support their daily well-being. Interviews will be conducted by the project’s research coordinator, a psychologist with specific training in qualitative methodology and semi-structured interviewing acquired during her master’s degree. A minority of interviews will be conducted by two master’s students, who will receive the same base training as well as additional targeted coaching from the research coordinator prior to data collection.

All participants enrolled in the project will be invited to take part in the qualitative interview; participation will be entirely voluntary, and no additional inclusion or exclusion criteria will be applied beyond enrolment in the main study.

#### Quantitative assessments

##### Baseline measures

Participants will be asked to complete an online battery of 12 questionnaires about sociodemographics and general health status, life events, spirituality, self-regulation and mental health. All measures will be administered online in French. All questionnaires possess good psychometric properties. The questionnaires not available in French language have been translated from English into French by native speakers well-experienced with clinical diagnostic instruments and back-translated by an English native speaker. Psychometric properties, including internal consistency and dimensional structure, will be evaluated in the study sample prior to their use in the main analyses. Individual response timing will automatically be registered to detect outliers in response behaviour. The collected EMA data will be stored in a secure web application hosted on the Lausanne University Hospital secured servers (REDCap^58 59^) directly on the completion of each baseline questionnaire or EMA survey, which will ensure the protection of data privacy.

Sociodemographic and general health status variables include questions on age, sex, parental education and profession, cultural background and family constellation as well as religious confession and history, belief in a single God or higher force that guides our destiny. Moreover, information will be collected on current and past mental healthcare utilisation, including consultations with mental health professionals and the use of prescribed psychotropic medication.

The Adverse Childhood Experience (ACE) section of the Paediatrics ACE and related life events screener (PEARLS)^60 61^ is a screening tool that assesses abuse, neglect and family dysfunction.

Concerning spirituality, we will use three self-report measures: (a) the Spirituality Scale (SS),^13^ which consists of four dimensions^62 63^: spiritual beliefs, self-discovery, self-awareness and collective consciousness, respect for others and the environment; (b) the Attitudes Related to Spirituality Scale,^64^ which focus on non-tautological aspects; (c) Finally, because meaning in life is considered a central existential dimension of spirituality in our conceptual framework, this dimension will be examined in greater depth using the MLQ.^16^ This instrument examines two dimensions of meaning in life: the presence of meaning (how full participants feel their lives are meaningful) and the search for meaning (how engaged and motivated participants are in efforts to find meaning or better understand the meaning in their lives).

Self-regulation will include: (a) the Cognitive Emotion Regulation Scale^65^ identifying adaptive and non-adaptive emotion regulation strategies; (b) trait self-control skills will be assessed with the French version of the Brief Self-Control Scale;^66^ (c) impulsivity will be assessed using the short French version of the Impulsive Behaviour Scale (UPPS)^67 68^ questionnaire that evaluate five impulsivity facets, including negative urgency, positive urgency, lack of premeditation, lack of perseverance and sensation seeking; (d) self-efficacy will be measured using the French adaptation of the General Self-Efficacy Scale (GSES),^69^ evaluating self-beliefs to cope with difficult demands in life.

Concerning mental health: the French version of the Achenbach System of Empirically Based Assessment, Child Behaviour Checklist (CBCL)—Youth Self-Report (YSR)^70 71^ will be used to assess behavioural and emotional problems, including eight categories: anxious/depressed, withdrawn/depressed, somatic complaints, social problems, thought problems, attention problems, rule-breaking behaviour and aggressive behaviour. The PMH Scale^72^ will be used to assess mental well-being.

The KidScreen-27^73–75^ will be used to assess five dimensions of QoL, including physical and psychological well-being, relationships with parents and autonomy, social support and peers and school environment.

##### Ecological momentary Assessment

Participants will receive a short training on how to answer questions from the EMA questionnaire. They will then answer the questions in the programmed questionnaire from their personal smartphone that prompts the participants at given time intervals. Participants will receive an SMS with a website link (within the REDCap environment^58 59^ where they will be able to answer the questions. Options to postpone the alarm/filling out the questions are available if participants are in a situation in which it is not possible to respond promptly. However, after a period of 1 hour it is not possible for the participants to respond to the survey anymore (to avoid retrospective assessments). Previous studies^76^ and our own experiences (mSanté study^77^ and ECOSREXT study^78^ have shown that the feasibility of EMA assessment is high (compliance above 70%). The average time to fill out the questionnaire each time is short (2–5 min).

More specifically, participants will be asked to answer the EMA surveys four times a day for 10 consecutive days at two different periods which will be 3 months apart: a morning survey (29–37 items before school/work), a noon and an afternoon survey (27–33 items after school/work), and an evening survey (38–45 items before bedtime). The surveys cover the domains of momentary context, emotional states, substance use, daily spirituality, self-control and impulsivity. The evening survey further includes questions on daily life events, QoL and overall health.

To minimise participant burden and maximise compliance during the EMA phases, several strategies were implemented. First, participant compensation was partly contingent on study compliance, with compensation adjusted according to the proportion of completed assessments. Second, the EMA platform incorporated gamification features designed to enhance engagement, including personalised avatars, visual icons, progress bars, colours and motivational messages. Finally, compliance was continuously monitored throughout the study. When repeated assessments were missed or a decline in participation was observed, participants were contacted by a member of the research team to identify potential difficulties, provide support and encourage continued engagement. Together, these procedures were designed to promote adherence while maintaining a positive participatory experience.

##### Follow-up assessments

Before the completion of the second EMA period, which is 3 months after the first EMA period, participants will receive the invitation to a survey. This consists of a follow-up of all the baseline questionnaires, except for the socio-demographics, the general health status and the PEARLS-ACE. We will replace the PEARLS-ACE with a non-validated questionnaire composed of three questions asking whether participants have experienced a major life event during the last 3 months (from the baseline assessment to the follow-up assessment) and ask them to specify the type of event and the time at which it occurred.

All the instruments that will be used throughout the study are summarised in table 2.

**Table 2:** Assessments

Occasions	Domains	Questionnaire
Qualitative assessment	Semistructured interview	Face-to-face or via Webex
Baseline assessment	Sociodemographics	Non-validated questionnaire
	General health status	Non-validated questionnaire
	Life events	ACE
	Spirituality	SS
		ARES
		MLQ
	Self-regulation	CERQ
		BSCS
		UPPS
		GSES
	Mental health	CBCL-YSR
		PMH-scale
	Quality of life	KidScreen-27
Follow-up assessment	Life events	Non-validated questionnaire
	Spirituality	SS
		ARES
		MLQ
	Self-regulation	CERQ
		BSCS
		UPPS
		GSES
	Mental health	CBCL-YSR
		PMH-Scale
	Quality of life	KidScreen-27
EMA (daily during 10 days for 2 periods)	Spirituality, self-control, impulsivity, emotional states, mental health, quality of life	Morning
		Noon
		Afternoon
		Evening
Final assessment	Satisfaction	Non-validated questionnaire

ACE, adverse childhood experience; ARES, Attitudes Related to Spirituality Scale; BSCS, Brief Self-Control Scale; CBCL-YSR, Child Behaviour Checklist–Youth Self-Report; CERQ, Cognitive Emotion Regulation Scale; EMA, Ecological Momentary Assessment; GSES, General Self-Efficacy Scale; MLQ, Meaning in Life Questionnaire; PMH-Scale, Positive Mental Health-Scale; SS, Spirituality Scale; UPPS, Impulsive Behaviour Scale.

### Statistical analysis plan

#### Data analysis

##### Qualitative approach

Transcription of verbal data will be analysed using reflexive thematic analysis method for identifying and reporting themes within data.^79^ Thematic analyses will be carried out by at least two experts and their results compared. Any differences between experts will be discussed to achieve a consensus. Consistent with recommendations for team-based reflexive thematic analysis, coding will be undertaken collaboratively by the project’s research coordinator and two master’s students involved in data collection; divergences in interpretation will be discussed and resolved through reflexive dialogue aimed at enriching the analysis.^80^ Sample size will not be determined by data saturation, as this concept is considered theoretically inconsistent with the interpretative and constructionist assumptions of reflexive thematic analysis.^80^ Instead, sample size will be guided by the principle of information power, considering the specificity of the study aim, the anticipated richness of participants’ accounts, and the diversity of youths, while remaining pragmatically bound by participant availability within the study timeframe.^81^ Data will be coded using MAXQDA.

##### Quantitative data

The analyses will be conducted using R^82^ software. Linear mixed models (LMMs) will be used to examine the lagged and cross-lagged associations between daily spirituality, daily self-control, momentary emotions and well-being, incorporating baseline (trait-level) measures of self-regulation and spirituality, and estimating within-person and between-person effects separately. Models will be estimated using data collected through EMA across two waves. All time-varying level-1 predictors are person-mean-centred so that within-person and between-person effects are not conflated. The autoregressive dependency in the outcome across occasions is modelled through a first-order autoregressive, AR(1), residual covariance structure. Below, the model is first specified for a single wave, and then extended to a pooled model spanning both waves:

Level 1 (within-person − WP):


$$\begin{array}{ll} WB_{it}= & \gamma_{0i}+\gamma_{1i}\;Spirituality\_PC_{i(t-1)}+\gamma_{2i}\;Self\;Control\_PC_{i(t-1)}+\\ \gamma_{3i}\;Emotions\_PC_{i(t-1)}+E_{it} \end{array}$$

Level 2 (between-person − BP):


$$\begin{array}{ll} \gamma_{0i}= & \beta 00+\beta_{01}QoL_{i}+\beta_{02}\;Mental\;Health_{i}+\beta_{03}\;Spirituality\_trait_{i}+\\ \beta_{04}\;Self\;Regulation\_trait_{i}+\beta_{05}\;Spirituality\_Mean_{i}+\\ \beta_{06}\;Self\;Control\_Mean_{i}+\beta_{07}\;Emotions\_Mean_{i}+R_{0i} \end{array}$$

where *WB* stands for daily well-being, *Spirituality_PC, Self Control_PC, and Emotions_PC* are the person-mean-centred daily EMA measures. There is no lagged well-being term; the autoregressive dependency is instead carried by the AR(1) structure on E_it_.

In addition :


$$\gamma 1i=\beta_{10}+R_{1i}\left(random\;slope:\;Spirituality\_P\right)$$

$$\gamma_{2i}=\beta_{20}+R_{2i}\;\left(random\;slope:Self\;Control\_PC\right)$$

$$\gamma_{3i}=\beta_{30}+R_{3i}\left(random\;slope:Emotions\_PC\right)$$

The index (it) indicates the observations for participant (i) and time (t), and (i(t–1)) refers to the observation for the same participant, same wave, at the previous assessment.

In the next step, both waves are pooled into a single estimation, with wave entered as a level-1 dummy (0=wave 1, 1=wave 2) that interacts with each within-person predictor. This lets every within-person effect differ by wave while still combining all occasions from both waves into one model. The formula for the pooled model is presented as:

Level 1 (within-person, both waves):


$$\begin{array}{ll} WB_{itw}= & \gamma_{0i}+\gamma_{1i}\;Spirituality\_PC_{i(t-1)w}+\gamma_{2i}\;SelfControl\_PC_{i(t-1)w}+\\ \gamma_{3i}\;Emotions\_PC_{i(t-1)w}+\gamma_{5}Wave_{itw}+\gamma_{6}{\left(Wave\times Spirituality\_PC\right)}_{i(t-1)w}+\\ \gamma_{7}{\left(Wave\times SelfControl\_PC\right)}_{i(t-1)w}+\gamma_{8}{\left(Wave\times Emotions\_PC\right)}_{i(t-1)w}+\\ E_{itw} \end{array}$$

The subscript (w) denotes the wave. Although the level-2 specification remains unchanged, the person-level means used to construct the level-2 ‘mean’ predictors are now calculated separately for each wave (i.e., a wave 1 mean and a wave 2 mean for each individual). These final parameter estimates (fixed effects, variance components, AR(1)) will be obtained using restricted maximum likelihood (REML). Any nested model comparisons on the fixed-effects structure (e.g., testing whether the Wave interaction terms improve fit via a likelihood-ratio test) will be refitted using maximum likelihood (ML), since REML likelihoods are not valid for comparing models that differ in fixed effects. Variance-component and random-effects structures (including comparisons of G structures, eg, unstructured vs diagonal) will be compared under REML, since these do not change the fixed-effects specification. Because this design requires an AR(1) residual correlation structure in combination with multiple correlated random slopes, the R package nlme is the most appropriate primary modelling framework. Specifically, lme() supports both an unstructured random-effects covariance matrix (pdSymm) and an AR(1) residual correlation structure (corAR1) within a single model specification.

Missing EMA data will not be deleted or imputed prior to model fitting. Because the LMM is estimated by (RE)ML rather than requiring a complete, balanced panel, each participant contributes all of their available occasions, and unequal numbers of observations across participants, including participants who completed only one of the two waves, are handled directly by the estimator under a missing-at-random (MAR) assumption, without listwise deletion of participants.

To exploit the full potential of this intensive longitudinal database, dynamic network analysis will be implemented by estimating a multilevel vector autoregression model on the EMA data^70^ to shed light on the evolution and interplay of well-being, spirituality, self-control and momentary emotions (measured 4×/day for 10 days). This will result in three different network architectures: (a) the temporal within-person network (lagged model, from one measure to the next one, but also across waves); (b) the contemporaneous within-person network (from a post hoc analysis) and (c) the between-person network structure.

The multilevel VAR will be estimated in R using the mlVAR package,^83^ which estimates the temporal, contemporaneous, and between-person networks simultaneously within a single multilevel modelling framework (correlated random-effects estimation, as opposed to fitting a separate first-order VAR per participant and aggregating afterwards). Temporal (lagged) paths will be estimated only within the same day, excluding the overnight lag between the last EMA prompt of 1 day and the first prompt of the next, since that gap is not comparable in duration to the within-day gaps between prompts; lagged paths will likewise not be estimated across the boundary between Wave 1 and Wave 2, given the extended interval separating the two waves. All variables will be person-mean-centred prior to estimation of the temporal and contemporaneous networks, consistent with the within-person centring approach used in the LMM models above, so that the resulting temporal and contemporaneous edges reflect within-person dynamics rather than between-person differences in average levels.

Missing EMA observations, expected given the burden of a 4×/day protocol over 10 days, will not require case deletion or imputation. Because mlVAR estimates the temporal and contemporaneous networks within a multilevel (mixed) modelling framework rather than fitting a separate, complete-case VAR per person, participants and occasions with missing beeps simply contribute fewer within-person lagged pairs to the likelihood, and all remaining available data are retained under an MAR assumption. The only missingness treated as structural rather than incidental is the overnight and between-wave gaps described above, which are excluded from the lagged design a priori rather than estimated as missing data.

### Sample size rationale

The power analysis is conducted only specifically for the primary outcome, by running 1000 simulations based on the LMM design with the following settings: (1) the interclass correlation coefficient was fixed at 0.75; (2) small effect sizes based on Cohen’s guidelines for standardised beta are considered at level (1) 0.21 between daily spirituality assessments and mental health. This analysis shows sufficient power (over 80%) to detect the effect of spirituality on daily well-being, with 10 days (one wave of 10 days) of observation across 100 patients at the 5% significance level. With a 20% rate of attrition recruitment of 120 participants is planned. All other planned analyses, including the second EMA wave, lagged and cross-lagged associations, multilevel vector autoregressive network analyses, analyses involving multiple predictors, and qualitative investigations, are exploratory in nature. These analyses are intended to identify potentially meaningful patterns and generate hypotheses for future research rather than to formally test a priori hypotheses.

## Patient and public involvement

We invited a panel of youths from the general population to provide feedback during the development phase of the project. They reviewed the qualitative semi-structured interview, the study procedure for the quantitative part of the study (i.e., length of EMA period and length of EMA questions), and the gamification elements planned for the EMA component (e.g., fun facts, choice of avatar) aimed at enhancing participant engagement and adherence. Moreover, their feedback was used to refine the spirituality-related interview questions, ensuring they were clear, concise and easy for participants to understand. We also asked the youths panel for input on how to report individual results back to participants. Finally, we intend to involve “patient and public involvement” partners in disseminating the study findings to the general public.

## Ethics and dissemination

### Ethics

This study adheres to the approved protocol, the Declaration of Helsinki, Good Clinical Practice guidelines, the Human Research Act (HRA), the Human Research Ordinance (HRO) and all applicable local regulations. Ethical approval was obtained from the Ethics Committee of the Vaud Canton (CER-VD, #2025-00088). All participants will receive oral and written information about the study, be given sufficient time to ask questions and provide written informed consent before participation.

### Dissemination

#### Publication

The findings from this study will be published in open-access peer-reviewed journals and presented at leading conferences on child and adolescent mental health, as well as meetings dedicated to ambulatory assessment. Results will also be shared with practitioners through the institute’s strong connections with clinics, educational institutions and third-sector organisations. Furthermore, we aim to make the outcomes accessible to the public via online and print media public events and social media platforms.

#### Data sharing policy

For participants who consent to the reuse of their data, de-identified data will be shared via a dedicated repository such as Zenodo. The main analyses will be preregistered on the Open Science Framework (OSF).

## Project’s impact

This study has the potential to make a significant impact in several areas. By adopting an innovative and original integrative approach, it will allow a fine-grained observation of the complex longitudinal dynamic associations between different components of spirituality, self-regulation and mental health. The findings of the study may help implement positive attitudes in adolescents and young adults’ relationships, life values, personal meaning or coping strategies.^84^ Moreover, the findings of the study may help to develop personalised preventive or therapeutic interventions, enhancing youths’ ability to cope with daily stress and increase their resilience through the reciprocal link between spirituality and positive or negative emotions. The study’s results may also raise awareness among the general population about the role of spirituality and self-regulation in mental health and how they can be used to improve well-being. The new and unexpected findings of this study may provide valuable insights for conducting larger-scale research with more a priori hypotheses in contrast to the more explorative nature of the current study.

## Supplementary material

10.1136/bmjopen-2026-116754 online supplemental file 1
