# Supplementary material for: Correlates between spirituality, self-regulation and mental health in youths from the community: A micro-longitudinal study protocol
Source: BMJ Open. 2026 Nov 11;16(11):e116754. doi: 10.1136/bmjopen-2026-116754 (PMC13756323; doi:10.1136/bmjopen-2026-116754)
Supplement: Supplementary file 1 [file bmjopen-2026-116754.reviewer_comments.pdf]

PEER REVIEW HISTORY

BMJ Open publishes all reviews undertaken for accepted manuscripts. Reviewers are asked to complete a checklist review form and are provided with free text boxes to elaborate on their assessment. These free text comments are reproduced below.

ARTICLE DETAILS

Title (Provisional)

Correlates between Spirituality, Self-Regulation and Mental Health in Youths from the Community: A Micro-Longitudinal Study Protocol

Authors

Urben, Sebastien; von Niederhäusern, Chloé; Ranjbar, Setareh; Plessen, Kerstin Jessica; Glaus, Jennifer

---

VERSION 1 - REVIEW

**Reviewer**	**1**
**Name**	**Rutenkröger, Mareike**
**Affiliation**	**University Hospital Hamburg-Eppendorf, Department of Medical Psychology**
**Date**	**25-Jul-2026**
**COI**	**none**

This study protocol presents an innovative mixed-methods investigation of the dynamic associations between spirituality, self-regulation, mental health, and quality of life among adolescents and young adults using ecological momentary assessment (EMA). The integration of qualitative interviews with intensive longitudinal data collection is a major strength and addresses an important gap in the current literature. The protocol is generally well written, the rationale is convincing, and the planned dissemination strategy is comprehensive. Nevertheless, several aspects require clarification before publication.

Major comments

1. Research questions and hypotheses require greater precision

Although the background thoroughly describes the theoretical framework, the specific research questions remain rather broad. The manuscript would benefit from explicitly stating primary and secondary hypotheses. Without clearly predefined hypotheses, the statistical analyses appear largely exploratory.

For example:

Which dimensions of spirituality are expected to predict mental health outcomes?

Is self-regulation hypothesized as a mediator, moderator, or parallel predictor?

Which outcomes are considered primary (well-being, psychopathology, quality of life)?

2. Sample size justification should account for the complete study design

The power analysis is based on one EMA wave and focuses on detecting the association between spirituality and well-being using LMM. However, the planned analyses include two EMA waves, lagged effects, cross-lagged associations,

multilevel vector autoregressive network analyses, multiple predictors, qualitative analyses.

It remains unclear whether the planned sample size is sufficient for these considerably more complex analyses. The authors should justify why the proposed sample is adequate for the most demanding planned analyses, particularly the dynamic network models.

3. Statistical analysis plan requires additional detail

The statistical analysis section would benefit from more methodological transparency.

How will missing EMA observations be handled?

Which software/packages will be used for the multilevel VAR analyses?

4. Burden of EMA participation

Participants complete four assessments per day over ten consecutive days in two separate waves. Some daily questionnaires contain up to 45 items. Although previous compliance rates are cited, the manuscript should discuss participant burden more explicitly. In particular: expected completion rate, strategies to minimize fatigue, handling of declining compliance over time, whether incentives depend on compliance.

5. Conceptual overlap between spirituality and meaning in life

Meaning in life is presented both as an inherent component of spirituality and as an independent construct measured separately (MLQ). The manuscript would benefit from explaining the conceptual distinction between these constructs and how overlap will be handled statistically (e.g., multicollinearity).

Minor comments

6. Inclusion criteria

The manuscript should clarify whether participants with current psychiatric diagnoses are eligible. Since mental health is one of the primary outcomes, information on psychiatric treatment or diagnosis would help characterize the sample.

7. Translation procedure

Several questionnaires were translated into French specifically for this study. Please clarify whether any psychometric validation or pilot testing of these translated versions is planned before use.

8. Qualitative methodology

The interview procedure is described only briefly. More information would strengthen the protocol, including sampling rationale, expected thematic saturation, interviewer training, coding software (e.g., NVivo, MAXQDA).

9. Language

The manuscript would benefit from careful language editing by a native English speaker. Several minor grammatical issues are present throughout the manuscript (e.g., "allow to examine" instead of "allows examining", inconsistent use of "youth", "youths", and "adolescents and young adults", occasional missing articles, and several long sentences that could be simplified).

VERSION 1 - AUTHOR RESPONSE

Reviewer 1: Dr. Mareike Rutenkröger, University Hospital Hamburg-Eppendorf

This study protocol presents an innovative mixed-methods investigation of the dynamic associations between spirituality, self-regulation, mental health, and quality of life among adolescents and young adults using ecological momentary assessment (EMA). The integration of qualitative interviews with intensive longitudinal data collection is a major strength and addresses an important gap in the current literature. The protocol is generally well written, the rationale is convincing, and the planned dissemination strategy is comprehensive. Nevertheless, several aspects require clarification before publication.

Authors' response: Thank you for this encouraging remark and the thoughtful comments. Below, you may find a point-by-point response to your concerns.

Major comments

1. Research questions and hypotheses require greater precision Although the background thoroughly describes the theoretical framework, the specific research questions remain rather broad. The manuscript would benefit from explicitly stating primary and secondary hypotheses. Without clearly predefined hypotheses, the statistical analyses appear largely exploratory.

For example:

Which dimensions of spirituality are expected to predict mental health outcomes? Is self-regulation hypothesized as a mediator, moderator, or parallel predictor? Which outcomes are considered primary (well-being, psychopathology, quality of life)?

Authors' response: We thank the Reviewer for this important comment. We agree that the hypotheses could be stated more explicitly. However, given the limited empirical literature simultaneously examining spirituality, self-regulation, and mental health in youth, the present study was, indeed, primarily exploratory in nature. Thus, our main objective was to investigate

whether spirituality contributes to the understanding of youth mental health beyond self-regulatory processes, rather than to test a priori hypotheses regarding the specific effects of specific components of spirituality or self-regulation dimensions.

Although previous research generally suggests positive associations between spirituality, adaptive self-regulation, and mental health, there is currently insufficient evidence to formulate precise hypotheses or specific roles (i.e., mediating or moderating) regarding the respective contributions of the different components of spirituality (e.g., presence vs. search for meaning) and self-regulation (e.g., self-control, emotion regulation, impulsivity, self-efficacy). Consequently, we adopted an exploratory approach to identify which dimensions were most strongly associated with the outcomes under investigation. However, the general direction of the expected link could be formulated. Thus, we adapted the current study section and we added (p. 10):

> *"Given the limited empirical evidence on the combined role of spirituality and self-regulation in youth mental health, the present study is primarily exploratory. Nevertheless, based on the few previous research, we might broadly expect that greater self-regulatory capacities would be associated with better mental health outcomes and that higher levels of spirituality, particularly the presence of meaning in life, would be positively related to positive mental health and psychological quality of life. The specific contributions and the role (i.e., mediating or moderating effect) of the different dimensions of spirituality and self-regulation will be explored without a priori hypotheses."*

Regarding the primary outcomes, mental health is considered as the primary outcome of interest. In particular, this concept includes positive aspects of mental health as well as psychopathology. To improve clarity, we have revised the Introduction. Now it reads (p. 4):

> *"Mental health should not be viewed solely as the absence of psychopathology (WHO, 2025) but rather as a multidimensional construct encompassing both well-being and psychological difficulties. Specifically, mental health can be conceptualized as consisting of two related yet distinct dimensions: well-being, including positive mental health (PMH) or psychological quality of life (QoL), and psychopathology (Keyes, 2005; OBSAN, 2025). Given that most studies have focused primarily on psychopathological symptoms, the present study adopts a more comprehensive approach by also examining the positive dimensions of mental health."*

2. Sample size justification should account for the complete study design The power analysis is based on one EMA wave and focuses on detecting the association between spirituality and well-being using LMM. However, the planned analyses include two EMA waves, lagged effects, cross-lagged associations, multilevel vector autoregressive

network analyses, multiple predictors, qualitative analyses. It remains unclear whether the planned sample size is sufficient for these considerably more complex analyses. The authors should justify why the proposed sample is adequate for the most demanding planned analyses, particularly the dynamic network models.

Authors' response: We thank the Reviewer for this important observation. We would like to clarify that the sample size calculation was conducted solely for the study's primary analyses, namely the association between self-regulation or spirituality and mental health during the first EMA wave. The study was explicitly powered to detect this effect using linear mixed-effects models.

All other planned analyses, including the second EMA wave, lagged and cross-lagged associations, multilevel vector autoregressive network analyses, dynamic network, analyses involving multiple predictors, and qualitative investigations, are exploratory in nature. These analyses are intended to identify potentially meaningful patterns and generate hypotheses for future research rather than to formally test a priori hypotheses. Consequently, no specific power calculations were performed for these secondary analyses, and any findings arising from them will be interpreted with appropriate caution.

To avoid ambiguity, we have revised this protocol to more clearly distinguish between the confirmatory and exploratory components of the study. Given the limited empirical literature on the joint role of self-regulation spirituality in youth mental health, we believe that these exploratory analyses can provide valuable insights and inform the design and power considerations of future confirmatory studies. Now it reads (p. 19):

> "*The power analysis is conducted only specifically for the primary outcome, by running 1000 simulations based on the LMM design with the following settings: (1) the interclass correlation coefficient (ICC) was fixed at 0.75; (2) small effect sizes based on Cohen's guidelines for standardized beta are considered at level (1) 0.21 between daily spirituality assessments and mental health. This analysis shows sufficient power (over 80%) to detect the effect of spirituality on daily well-being, with 10 days (one wave of 10 days) of observation across 100 patients at the 5% significance level. With 20% rate of attrition recruitment of 120 participants are planned. All other planned analyses, including the second EMA wave, lagged and cross-lagged associations, multilevel vector autoregressive network analyses, analyses involving multiple predictors, and qualitative investigations, are exploratory in nature. These analyses are intended to identify potentially meaningful patterns and generate hypotheses for future research rather than to formally test a priori hypotheses.*"

3. Statistical analysis plan requires additional detail

The statistical analysis section would benefit from more methodological transparency. How will missing EMA observations be handled? Which software/packages will be used for the multilevel VAR analyses?

Authors' response: Accordingly, we added more information in the statistical analysis plan. Now it reads (pp. 15-19):

> *"The analyses will be conducted using R[84] software. Linear Mixed Models (LMM) will be used to examine the lagged and cross-lagged associations between daily spirituality, daily self-control, momentary emotions, and well-being, incorporating baseline (trait-level) measures of self-regulation and spirituality, and estimating within-person and between-person effects separately. Models will be estimated using data collected through EMA across two waves. All time-varying Level-1 predictors are person-mean-centered so that within-person and between-person effects are not conflated. The autoregressive dependency in the outcome across occasions is modeled through a first-order autoregressive, AR(1), residual covariance structure. Below, the model is first specified for a single wave, and then extended to a pooled model spanning both waves:*
>
> *Level 1 (within-person - WP):*
>
> $WB_{it} = \gamma_{0i} + \gamma_{1i}\ Spirituality\_PC_{i(t-1)} + \gamma_{2i}\ Self\ Control\_PC_{i(t-1)} + \gamma_{3i}\ Emotions\_PC_{i(t-1)} + E_{it}$
>
> *Level 2 (between-person - BP):*
>
> $\gamma_{0i} = \beta 00 + \beta_{01}\ Quality\ of\ Life_i + \beta_{02}\ Mental\ \ Health_i + \beta_{03}\ Spirituality\_trait_i + \beta_{04}\ Self\ Regulation\_trait_i + \beta_{05}\ Spirituality\_Mean_i + \beta_{06}\ Self\ Control\_Mean_i + \beta_{07}\ Emotions\_Mean_i + R_{0i},$
>
> *where WB stands for daily well-being, Spirituality_PC, Self Control_PC, and Emotions_PC are the person-mean-centered daily EMA measures. There is no lagged well-being term; the autoregressive dependency is instead carried by the AR(1) structure on* E_{it}.
>
> *In addition :*
>
> $\gamma_{1i} = \beta_{10} + R_{1i}$ *(random slope: Spirituality_PC)*
>
> $\gamma_{2i} = \beta_{20} + R_{2i}$ *(random slope: Self Control_PC)*
>
> $\gamma_{3i} = \beta_{30} + R_{3i}$ *(random slope: Emotions_PC)*

The index (it) indicates the observations for participant (i) and time (t), and (i(t-1)) refers to the observation for the same participant, same wave, at the previous assessment.

In the next step both waves are pooled into a single estimation, with Wave entered as a Level-1 dummy (0 = Wave 1, 1 = Wave 2) that interacts with each within-person predictor. This lets every within-person effect differ by wave while still combining all occasions from both waves into one model. The formula for the pooled model is presented as:

Level 1 (within-person, both waves):

$$WB_{itw} = \gamma_{0i} + \gamma_{1i}\ Spirituality\_PC_{i(t-1)w} + \gamma_{2i}\ SelfControl\_PC_{i(t-1)w} + \gamma_{3i}\ Emotions\_PC_{i(t-1)w} + \gamma_5\ Wave_{itw} + \gamma_6\ (Wave \times Spirituality\_PC)_{i(t-1)w} + \gamma_7\ (Wave \times SelfControl\_PC)_{i(t-1)w} + \gamma_8\ (Wave \times Emotions\_PC)_{i(t-1)w} + E_{itw}$$

The subscript (w) denotes the wave. Although the Level-2 specification remains unchanged, the person-level means used to construct the Level-2 "Mean" predictors are now calculated separately for each wave (i.e., a Wave 1 mean and a Wave 2 mean for each individual). This Final parameter estimates (fixed effects, variance components, AR(1) will be obtained using Restricted Maximum Likelihood (REML). Any nested model comparisons on the fixed-effects structure (e.g., testing whether the Wave interaction terms improve fit via a likelihood-ratio test) will be refit using Maximum Likelihood (ML), since REML likelihoods are not valid for comparing models that differ in fixed effects. Variance-component and random-effects structures (including comparisons of G structures, e.g., unstructured vs. diagonal) will be compared under REML, since these do not change the fixed-effects specification. Because this design requires an AR(1) residual correlation structure in combination with multiple correlated random slopes, the R package nlme is the most appropriate primary modeling framework. Specifically, lme() supports both an unstructured random-effects covariance matrix (pdSymm) and an AR(1) residual correlation structure (corAR1) within a single model specification.

Missing EMA data will not be deleted or imputed prior to model fitting. Because the LMM is estimated by (RE)ML rather than requiring a complete, balanced panel, each participant contributes all of their available occasions, and unequal numbers of observations across participants, including participants who completed only one of the two waves, are handled directly by the estimator under a missing-at-random (MAR) assumption, without listwise deletion of participants.

To exploit the full potential of this intensive longitudinal database, dynamic network analysis will be implemented by estimating a multilevel vector autoregression model on the EMA data[71] to shed light on the evolution and interplay of well-being, spirituality, self-control, and momentary emotions (measured 4x/day for 10 days). This will result in three different network architectures: (a) the temporal within-person network (lagged model, from one measure to the next one, but also across waves); (b) the contemporaneous within-person network (from a post-hoc analysis); (c) the between-person network structure.

The multilevel VAR will be estimated in R using the mlVAR package[83], which estimates the temporal, contemporaneous, and between-person networks simultaneously within a single multilevel modeling framework (correlated random-effects estimation, as opposed to fitting a separate first-order VAR per participant and aggregating afterward). Temporal (lagged) paths will be estimated only within the same day, excluding the overnight lag between the last EMA prompt of one day and the first prompt of the next, since that gap is not comparable in duration to the within-day gaps between prompts; lagged paths will likewise not be estimated across the boundary between Wave 1 and Wave 2, given the extended interval separating the two waves. All variables will be person-mean-centered prior to estimation of the temporal and contemporaneous networks, consistent with the within-person centering approach used in the LMM models above, so that the resulting temporal and contemporaneous edges reflect within-person dynamics rather than between-person differences in average levels.

Missing EMA observations, expected given the burden of a 4x/day protocol over 10 days, will not require case deletion or imputation. Because mlVAR estimates the temporal and contemporaneous networks within a multilevel (mixed) modeling framework rather than fitting a separate, complete-case VAR per person, participants and occasions with missing beeps simply contribute fewer within-person lagged pairs to the likelihood, and all remaining available data are retained under a missing-at-random (MAR) assumption. The only missingness treated as structural rather than incidental is the overnight and between-wave gaps described above, which are excluded from the lagged design a priori rather than estimated as missing data."

4. Burden of EMA participation

Participants complete four assessments per day over ten consecutive days in two separate waves. Some daily questionnaires contain up to 45 items. Although previous compliance rates are cited, the manuscript should discuss participant burden more explicitly. In particular:

expected completion rate, strategies to minimize fatigue, handling of declining compliance over time, whether incentives depend on compliance.

Authors' response: We thank the Reviewer for this comment and agree that participant burden deserves further discussion. Several elements were incorporated into the protocol to maximize engagement and minimize fatigue. First, participants receive compensation proportional to their level of participation, with up to CHF 150 distributed across the different phases of the study (i.e., interview, questionnaires, and EMA). Eligibility for compensation requires completion of at least two-thirds of the questionnaires during each assessment period of EMA. This threshold was chosen based on recommendations for intensive longitudinal studies, balancing data quality considerations with the recognition that perfect compliance is unrealistic in repeated assessment designs.

Second, the EMA protocol was developed in collaboration with adolescents and young adults through a patient and public involvement and engagement (PPIE) process. Particular attention was paid to user experience. The surveys include several gamification features, such as personalized avatars, visual icons, progress indicators, colors, and encouraging messages, which were specifically implemented to make the repeated assessments more enjoyable and reduce fatigue.

Third, participant engagement is actively monitored throughout the study. When compliance begins to decline or questionnaires are repeatedly missed, participants are contacted by a member of the research team to identify potential difficulties, provide support, and encourage continued participation.

Importantly, data from the first phase of the project suggest that participant burden is manageable. Among the participants enrolled, compliance with the EMA protocol exceeded 95% at baseline (95.5%) and remained very high at follow-up (94.3%), despite the intensive nature of the assessment schedule. These findings indicate excellent acceptability of the protocol and suggest that the procedures implemented to support engagement were effective in maintaining participation over time.

To address the Reviewer's concern, we have added a paragraph to the manuscript describing the compensation procedure, engagement strategies, management of declining compliance. Now it reads (p. 14):

> *"To minimize participant burden and maximize compliance during the EMA phases, several strategies were implemented. First, participant compensation was partly contingent on study compliance, with compensation adjusted according to the proportion of completed components of the study (i.e., qualitative interview, baseline questionnaires, EMA) assessments. Second, the EMA platform incorporated gamification features designed to enhance engagement, including personalized avatars, visual icons, progress bars, colors, and motivational messages. Finally, compliance was continuously monitored throughout the study. When repeated assessments were missed or a decline in participation was observed, participants were*

contacted by a member of the research team to identify potential difficulties, provide support, and encourage continued engagement. Together, these procedures were designed to promote adherence while maintaining a positive participant experience."

5. Conceptual overlap between spirituality and meaning in life
Meaning in life is presented both as an inherent component of spirituality and as an independent construct measured separately (MLQ). The manuscript would benefit from explaining the conceptual distinction between these constructs and how overlap will be handled statistically (e.g., multicollinearity).

Authors' response: Thank you for this important observation. We agree that spirituality and meaning in life are conceptually related and can overlap, particularly because several influential definitions of spirituality include meaning and purpose among their core attributes (Lalani, 2020; Panzini et al., 2017). In the revised manuscript, we will clarify that spirituality is treated as a broader construct encompassing not only meaning in life but also transcendence, connectedness, and existential orientation, whereas meaning in life is treated as a more specific psychological construct referring to the perceived presence of meaning and the search for meaning as assessed by the MLQ (Jovanovic et al., 2025; Lalani, 2020; Steger et al., 2006).

Empirically, prior studies indicate that these constructs are not redundant, and that meaning in life often shows unique associations with well-being beyond religious/spiritual variables (Aglozo et al., 2021; Yoon et al., 2021). To address potential overlap statistically, we will report their bivariate association and examine collinearity diagnostics.

In the manuscript, we added this information. Now it reads (p. 5):

> *"Thus, spirituality is treated as a broader construct encompassing transcendence, connectedness, and existential orientation, whereas meaning in life is treated as a specific psychological construct referring to the perceived presence of meaning and the search for meaning as assessed by the Meaning in Life Questionnaire (MLQ) (Jovanovic et al., 2025; Lalani, 2020; Panzini et al., 2017; Steger et al., 2006; Yoon et al., 2021)."*

In the Method section we introduce also more specifically the MLQ (p. 12):

> *"Finally, because meaning in life is considered a central existential dimension of spirituality in our conceptual framework, this dimension will be examined in greater depth using the Meaning in Life Questionnaire (MLQ). This instrument examines two dimensions of meaning in life:…"*

Minor comments

6. Inclusion criteria

The manuscript should clarify whether participants with current psychiatric diagnoses are eligible. Since mental health is one of the primary outcomes, information on psychiatric treatment or diagnosis would help characterize the sample.

Authors' response: We thank the Reviewer for this comment. Participants with current or past psychiatric disorders are not excluded from the study. The inclusion criteria are intentionally broad, as the project aims to investigate mental health across a continuum ranging from well-being to psychological difficulties in a community, population-based sample of youth.

To better characterize the sample, socio-demographic data include information on both current and past mental health care utilization. Specifically, participants are asked whether they have previously consulted, or are currently consulting, a mental health professional (e.g., psychiatrist, psychologist, psychotherapist) and whether they are currently prescribed psychotropic medication. These variables will allow us to provide a detailed description of the sample and to account for mental health treatment history when relevant.

To improve clarity, we have revised the manuscript to explicitly state that participants with current psychiatric diagnoses are eligible and to indicate that information on mental health consultations and prescribed medication is collected as part of the socio-demographic assessment. Now it reads (p. 10):

> *"Participants with current or past psychiatric disorders were eligible for inclusion. Consistent with the study's objective of examining mental health across the continuum from well-being to psychological difficulties, no exclusion criteria related to psychiatric diagnoses were applied.*

And (p. 12):

> *Moreover, information on current and past mental health care utilization, including consultations with mental health professionals and the use of prescribed psychotropic medication."*

7. Translation procedure

Several questionnaires were translated into French specifically for this study. Please clarify whether any psychometric validation or pilot testing of these translated versions is planned before use.

Authors' response: We thank the Reviewer for this important comment. The Spirituality Scale (SS), Attitudes Related to Spirituality Scale (ARES), Positive Mental Health (PMH) Scale, and the daily spirituality items in the EMA, were translated into French using a forward-backward translation procedure conducted by native speakers. While a separate formal validation study is not planned, we agree that the psychometric performance of these translated versions should be documented. We have therefore clarified in the manuscript that reliability indices and factor

structure analyses will be examined within the study sample prior to substantive analyses to ensure the adequacy of the French versions.

We have now added this information in the methods part as follows (p. 12):

> "*Psychometric properties, including internal consistency and dimensional structure, will be evaluated in the study sample prior to their use in the main analyses.*"

8. Qualitative methodology

The interview procedure is described only briefly. More information would strengthen the protocol, including sampling rationale, expected thematic saturation, interviewer training, coding software (e.g., NVivo, MAXQDA).

Authors' response: We thank the Reviewer for this comment. We have added information on the interviewer training, the sampling rationale, and the coding software in section 2.3.1, and on the coding process in section 2.4.1. Regarding thematic saturation, we clarified that sample size is guided by the principle of information power (Malterud et al., 2016) rather than data saturation, as the latter is considered theoretically inconsistent with the reflexive thematic analysis approach adopted in this study (Braun & Clarke, 2019).

In the measure section we added (p. 11):

> *"Interviews will be conducted by the project's research coordinator, a psychologist with specific training in qualitative methodology and semi-structured interviewing acquired during her master's degree. A minority of interviews will be conducted by two master's students, who will receive the same base training as well as additional targeted coaching from the research coordinator prior to data collection.*
>
> *All participants enrolled in the project will be invited to take part in the qualitative interview; participation will be entirely voluntary and no additional inclusion or exclusion criteria will be applied beyond enrolment in the main study."*

And in the data analyses section (p. 15):

> *"Transcription of verbal data will be analyzed using reflexive thematic analysis method for identifying and reporting themes within data (Braun & Clarke, 2006). Consistent with recommendations for team-based reflexive thematic analysis, coding will be undertaken collaboratively by the project's research coordinator and two master's students involved in data collection; divergences in interpretation will be discussed and resolved through reflexive dialogue aimed at enriching the analysis (Braun & Clarke, 2021). Sample size will not be determined by data saturation, as this concept is considered theoretically inconsistent with the interpretative and constructionist*

assumptions of reflexive thematic analysis (Braun & Clarke, 2021). Instead, sample size will be guided by the principle of information power, considering the specificity of the study aim, the anticipated richness of participants' accounts, and the diversity of the SPIRIT cohort, while remaining pragmatically bound by participant availability within the study timeframe (Malterud et al., 2016). Data will be coded using MAXQDA."

9. Language

The manuscript would benefit from careful language editing by a native English speaker. Several minor grammatical issues are present throughout the manuscript (e.g., "allow to examine" instead of "allows examining", inconsistent use of "youth", "youths", and "adolescents and young adults", occasional missing articles, and several long sentences that could be simplified).

Authors' response: Accordingly, we carefully proofread the manuscript and make the appropriate changes.

References

Aglozo, E. Y., Akotia, C. S., Osei-Tutu, A., & Annor, F. (2021). Spirituality and subjective well-being among Ghanaian older adults: optimism and meaning in life as mediators. *Aging & Mental Health*, *25*(2), 306–315. https://doi.org/10.1080/13607863.2019.1697203

Braun, V., & Clarke, V. (2006). Using thematic analysis in psychology. *Qualitative Research in Psychology*, *3*(2), 77–101. https://doi.org/10.1191/1478088706qp063oa

Braun, V., & Clarke, V. (2021). To saturate or not to saturate? Questioning data saturation as a useful concept for thematic analysis and sample-size rationales. *Qualitative Research in Sport Exercise and Health*, *13*(2), 201–216. https://doi.org/10.1080/2159676x.2019.1704846

Epskamp, S., Deserno, M., & Bringmann, L. (2026). mlVAR: Multi-Level Vector Autoregression. *R package version 0.7.3*.

Huijs, T., Braam, A. W., Kruizinga, R., Jacobs, N., Reijnders, J., & Simons, M. (2024). Spirituality as a Predictor of Well-Being, Mental Distress or Both: A Four-Week Follow-Up Study in a Sample of Dutch and Belgian Adults. *Religions*, *15*(2). https://doi.org/10.3390/rel15020179

Jovanovic, V., Ilic, M., Sakan, D., & Brdar, I. (2025). The Meaning in Life Questionnaire: Revisiting the Evidence of Validity and Measurement Invariance Using the Exploratory Structural Equation Modeling. *Assessment*, *32*(8), 1274–1292. https://doi.org/10.1177/10731911241304223

Keyes, C. L. (2005). Mental illness and/or mental health? Investigating axioms of the complete state model of health. *Journal of consulting and clinical psychology*, *73*, 539.

Lalani, N. (2020). Meanings and Interpretations of Spirituality in Nursing and Health. *Religions*, *11*(9). https://doi.org/10.3390/rel11090428

Malterud, K., Siersma, V. D., & Guassora, A. D. (2016). Sample Size in Qualitative Interview Studies: Guided by Information Power. *Qualitative Health Research*, *26*(13), 1753–1760. https://doi.org/10.1177/1049732315617444

OBSAN. (2025). *Santé mentale en Suisse: évolution, promotion, prévention et prise en charge*. OFS.

Panzini, R. G., Mosqueiro, B. P., Zimpel, R. R., Bandeira, D. R., Rocha, N. S., & Fleck, M. P. (2017). Quality-of-life and spirituality. *International Review of Psychiatry*, *29*(3), 263–282. https://doi.org/10.1080/09540261.2017.1285553

Steger, M. F., Frazier, P., Oishi, S., & Kaler, M. (2006). The meaning in life questionnaire: Assessing the presence of and search for meaning in life. *Journal of Counseling Psychology*, *53*(1), 80–93. https://doi.org/10.1037/0022-0167.53.1.80

WHO. (2025). https://www.who.int/news-room/fact-sheets/detail/mental-health-strengthening-our-response

Yoon, E., Cabirou, L., Hoepf, A., & Knoll, M. (2021). Interrelations of religiousness/spirituality, meaning in life, and mental health. *Counselling Psychology Quarterly*, *34*, 219–234. https://doi.org/10.1080/09515070.2020.1712651

VERSION 2 - REVIEW

Reviewer 1

Name Rutenkröger, Mareike

Affiliation University Hospital Hamburg-Eppendorf, Department of Medical Psychology

Date 13-Oct-2026

COI

Thank you for revising the mansucript based on my suggestions, I think it is fit for publicaiton now.
