# Supplementary material for: Correlates between spirituality, self-regulation and mental health in youths from the community: A micro-longitudinal study protocol
Source: BMJ Open. 2026 Nov 11;16(11):e116754. doi: 10.1136/bmjopen-2026-116754 (PMC13756323; doi:10.1136/bmjopen-2026-116754)
Supplement: Supplementary file 2 [file bmjopen-16-11-s001.docx]

Supplementary file 1

**Qualitative Assessment - Semi-Structured Interview**

0. What gives meaning to your life?

1. Definition of spirituality.

What do you understand by spirituality?

Is it different from religion?

2. What is your current religious affiliation?

Please specify the denomination or faith tradition.

If it has changed, what was your previous religious affiliation?

3. What are your current beliefs? Or do you have any other beliefs?

Please specify your spiritual and/or religious beliefs.

If they have changed, what were your previous beliefs?

4. Have you experienced changes in your spiritual beliefs during your life?

If yes, what changes occurred and under what circumstances?

5. Do you participate in religious services or religious/spiritual activities with other people?

If yes, which activities and how often?

If your participation has changed over time, did you previously attend religious services or group activities related to your spirituality?

6. Do you engage in individual spiritual or religious practices, such as prayer, meditation, reading related to your faith, or other practices?

If yes, which practices and how often?

7. To what extent do your beliefs give meaning to your life?

To what extent do your beliefs provide you with resources, strength, or support?

Do your beliefs or religious affiliations create any difficulties or challenges for you?

8. Do you have role models, examples, or figures you follow (living or deceased)?

Are there role models, examples, or figures who inspire you?

Are there role models, examples, or figures who influence the way you think about life?

Are there role models, examples, or figures who influence the way you behave or act?
